# Effect of cevimeline and different concentration of gum arabic on parotid salivary gland function in methotrexate-induced xerostomia: a comparative study

**DOI:** 10.1186/s12903-024-04374-8

**Published:** 2024-05-28

**Authors:** Mahmoud Mohamed Aboulfotoh

**Affiliations:** Oral Biology Department, Faculty of Oral and Dental Medicine, Delta University for Science and Technology, Gamasa, Egypt

**Keywords:** Cevimeline, Gum arabic, Methotrexate, Parotid, Immunohistochemical staining

## Abstract

**Objective:**

This study assessed the effect of cevimeline and different concentrations of gum arabic on the parotid gland of rats being given xerostomia-inducing methotrexate.

**Methods:**

One hundred twenty-five rats were divided into five equal groups of twenty-five each. The rats in Group I received basic diets, while those in Groups II, III, IV, and V received 20 mg/kg MTX as a single intraperitoneal dose on day one. Group III received 10 mg/kg CVM dissolved in saline orally and daily, and the other two groups ^received a 10% W/V^ aqueous suspension of GA. Therefore, Group IV received 2 ml/kg suspension orally and daily, while Group V received 3 ml/kg suspension orally and daily. After 9 days, the parotid glands were dissected carefully and prepared for hematoxylin and eosin (H&E) staining as a routine histological stain and caspase-3 and Ki67 immunohistochemical staining. Quantitative data from α-Caspase-3 staining and Ki67 staining were statistically analysed using one-way ANOVA followed by Tukey’s multiple comparisons post hoc test.

**Results:**

Regarding caspase-3 and Ki67 immunohistochemical staining, one-way ANOVA revealed a significant difference among the five groups. For Caspase-3, the highest mean value was for group II (54.21 ± 6.90), and the lowest mean value was for group I (15.75 ± 3.67). The other three groups had mean values of 31.09 ± 5.90, 30.76 ± 5.82, and 20.65 ± 3.47 for groups III, IV, and V, respectively. For Ki67, the highest mean value was for group I (61.70 ± 6.58), and the lowest value was for group II (18.14a ± 5.16). The other three groups had mean values of 34.4 ± 9.27, 48.03 ± 8.40, and 50.63 ± 8.27 for groups III, IV, and V, respectively.

**Conclusion:**

GA, rather than the normally used drug CVM, had a desirable effect on the salivary glands of patients with xerostomia.

## Introduction

Maintaining the integrity of oral tissue depends critically on saliva, which is released by the acini of the salivary glands and controlled by duct cells. Among its various constituents, it provides lubrication, digestion, and enamel preservation, as well as antibacterial, antifungal, and anti-inflammatory properties [1]. 90% of saliva generated daily is secreted by the primary salivary glands, with the remaining 25% being produced by the parotid gland [2]. On the other hand, the subjective sensation of dry mouth, or xerostomia, can be caused by various illnesses, such as bone marrow transplants, radiation treatment for head and neck malignancies, and Sjögren’s syndrome [3]. However, severe oral dryness results from the destruction of salivary gland tissue and leads to loss of buffering action and salivary electrolytes [2].

Therefore, a decrease in salivation alters the quality of life of patients because it affects swallowing, mastication, and speech disturbance [4]. Therefore, the only medications that have been approved by the FDA for the treatment of xerostomia are pilocarpine and cevimeline (CVM), as they are used for the treatment of xerostomia and dry eye caused by head and neck cancer and Sjögren’s syndrome [3]. CVM is a derivative of azaspirodecan, which functions as an analogue of acetylcholine; it operates specifically on M1- and M3-muscarinic receptors and has a greater affinity for M3. Because of its pharmacological action, more tears and saliva are secreted [5]. Braga et al. [6] reported that CVM had an advantage over pilocarpine, which had longer induced salivation and a longer elimination half-life in the human body.

However, both pilocarpine and CVM have numerous negative effects, including diarrhea, trembling, increased perspiration, and anxiety [7]. Consequently, there is a critical need for further effective therapies of organic origin.

GA is the dried gummy exudate from mature Acacia senegal and Acacia seyal trees, which are primarily found in Sudan’s African region. It is considered a food additive and has played a significant biological role in reducing plasma cholesterol levels, exerting anticarcinogenic effects [8], and exerting antioxidant effects on animals and humans [9] since ancient times [10].

In 1974, the FDA considered GA a food additive and accepted it as a safe food with specific limitations [11] because it is rich in fiber and edible [12]. However, studies have proposed that in patients with chronic kidney disease, receiving fiber improves kidney health [13]. However, this study investigated the effect of GA on improving the parotid salivary gland in rats with xerostomia caused by methotrexate (MTX) compared to that of CVM, which is a normal drug on the market.

## Materials and methods

### Sample size calculation

The statistical program G power version 3.1 (Franz Faul) was used to determine the sample size. The sample size was calculated using fixed-effects, omnibus, 1-way analysis of variance (ANOVA) given α, power, and effect size. Five groups, an effect size of 0.4, a power of 0.95, and an α error probability of 0.05, were the input values. The results showed that 125 rats should be the minimum sample size.

### Materials and chemicals

Methotrexate (MTX) was obtained from Hospira Company (Maidenhead, UK) as a vial for injection, each containing 50 mg/5 ml injectable solution. Cevimeline was obtained from Nippon Kayaku Co., Ltd. (Tokyo, Japan), and each capsule contained 30 mg of Cevimeline. Finally, GA is obtained from the local market as a powder that is soluble in water.

### Experimental protocol

The Delta University for Science and Technology animal house served as the venue for this experiment. The Ethics Committee of the Faculty of Dentistry at Delta University for Science and Technology approved the experiment, which was carried out according to the Animal Research: Reporting of In Vivo Experiments (ARRIVE) protocols (FODMRC-2023-00100). In this study, 125 albino rats weighing 180 ± 20 g and aged between 12 and 14 weeks were acquired from the Ministry of Health in Egypt’s Vaccine and Immunity Organization. The animals were kept in conventional settings, with well-ventilated cages of an appropriate size, a 12/12 light/dark cycle, a temperature of 23 ± 2 °C, and 60% humidity. The rats had unlimited access to water and were fed a baseline diet. After one week of acclimation, the animals were randomly divided into 5 groups, with 25 animals in each group and only 8 to 9 rats in each cage. Group I, the control group included 25 rats that received a basic diet for 9 days; group II, the MTX group included 25 rats that received 20 mg/kg MTX as a single intraperitoneal dose on day one and were maintained under observation for 9 days [14]; group III, the MTX + CVM group included 25 rats that received MTX at the same dose in the control group and Cevimeline 10 mg/kg dissolved in saline taken orally daily for 9 days [15]; group IV, the MTX + Gum2 group included 25 rats that received MTX at the same dose in the control group and ^a 10% W/V^ aqueous suspension of gum Arabic powder (2 mL/kg) taken orally for 9 days [16]; group V, the MTX + Gum3 group included 25 rats that received MTX at the same dose in the control group and ^a 10% W/V^ aqueous suspension of gum Arabic powder (3 mL/kg) taken orally for 9 days [17].

### Rat euthanasia and tissue preparation

After 9 days, the rats were meticulously dissected, the head skin was cut from the side of the ear, and an overdose of phenobarbital sodium salt was used to euthanize them. The parotid gland was dissected for staining with hematoxylin and eosin (H&E) as a routine stain, caspase 3 immunohistochemical marker as an apoptotic marker, and antigen Kiel 67 (Ki67) immunohistochemical marker for cellular proliferation. After being fixed for one day in 10% neutral-buffered formalin, the parotid specimens were cleaned in xylene, dehydrated in increasing alcohol grades, and embedded in soft paraffin. All staining was performed according to a guide for each marker preparation.

A digital camera (VE-MC5 5.0 MP) mounted on an Olympus microscope with a magnification lens was used to take pictures of the slides. The final photos were processed with Fiji ImageJ (version 1.51r; NIH, Maryland, USA) on an Intel® core I7® computer. Using the color deconvolution plugin, the percentage of stained area was calculated. Five random fields on each slide were examined.

### Statistical analysis

Quantitative Caspase-3 and Ki67 immunostaining data were statistically analysed using one-way ANOVA with GraphPad Prism (version 8.0.0 for Windows, GraphPad Software, San Diego, CA, USA, www.graphpad.com, accessed on 21 April 2022), followed by Tukey’s multiple comparisons post hoc test. The data are presented as the mean ± standard deviation of the mean, and significance was considered at *p* < 0.05.

## Results

### Hematoxylin and eosin staining results

**Fig. 1:**
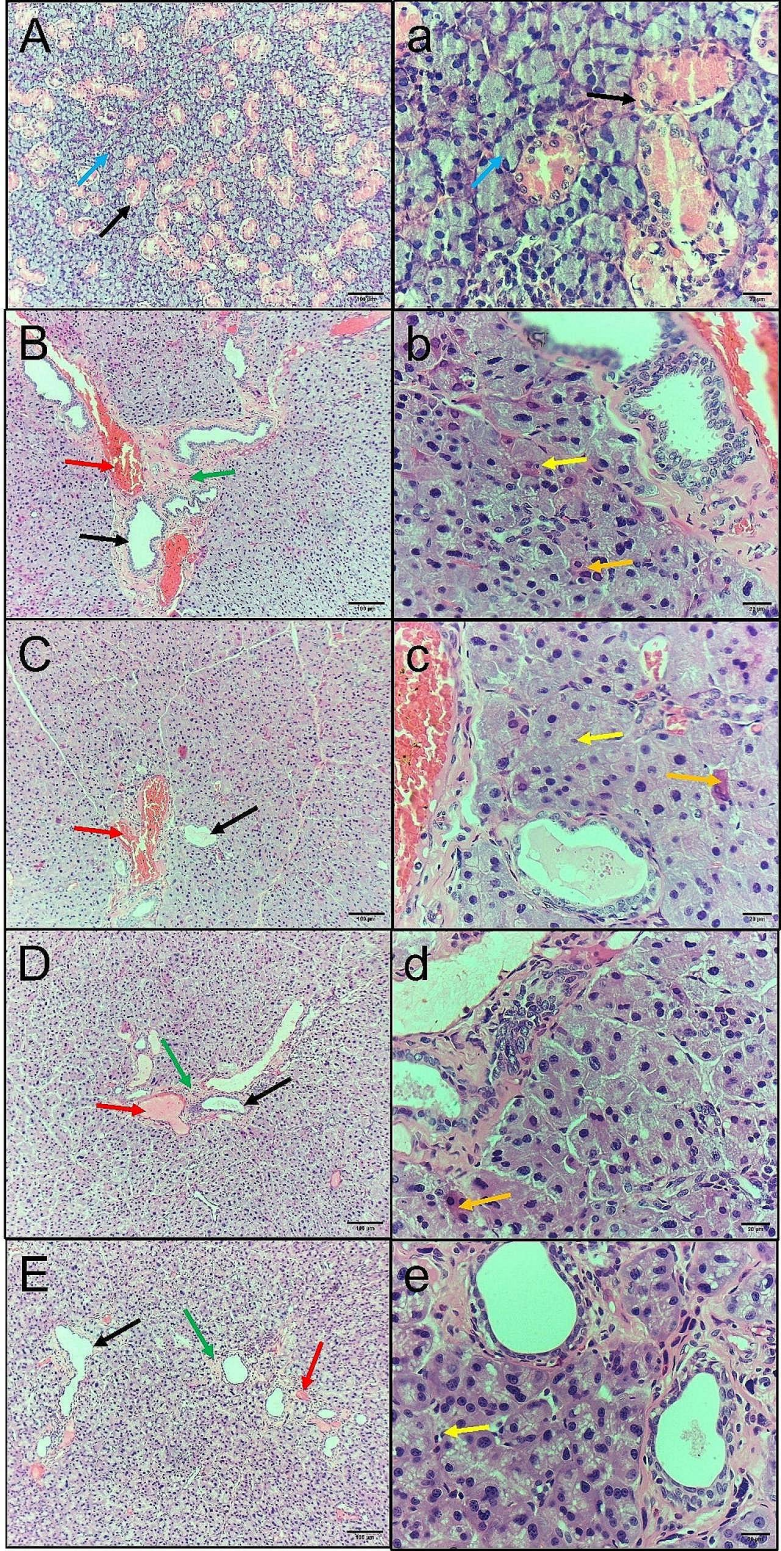
Soft paraffin section showing the control group **(A, a)** with normal acini **(blue arrow)**, marked ductal dilation **(black arrow)**, and normal duct shape **(black arrow)**; **(B, b)** The MTX group showing periductal fibrosis **(green arrow)**, dilated congested blood vessels **(red arrow)** and degeneration of acinar cells **(yellow arrow)**; **(C, c**) The MTX + CVM group showing mild ductal dilation **(black arrow)**, dilated congested blood vessels **(red arrow**) and degeneration of acinar cells **(yellow arrow)**, few pyknotic nuclei **(orange arrow)**; **(D, d)** The MTX + Gum2 group showing moderate dilation of the ducts **(black arrow)**, periductal fibrosis **(green arrow)**, mildly congested blood vessels **(red arrow)**, minimal acinar degeneration **(yellow arrow)** and few pyknotic nuclei **(orange arrow)**; **(E, e)** The MTX + Gum3 group showing minimal dilation of the ducts **(black arrow)**, minimal periductal fibrosis **(green arrow)**, mildly congested blood vessels **(red arrow)** and minimal acinar degeneration **(yellow arrow)**

The control group exhibited a normal salivary gland structure with salivary ducts and closely packed serous acini formed of pyramidal-shaped cells with rounded basal nuclei (Fig. 1A, a). In the MTX group, intense dilatation of the ducts, periductal fibrosis, dilated congested blood vessels, degeneration of acinar cells, and shrunken pyknotic nuclei were observed (Fig. 1B, b). The MTX + CVM group exhibited mild ductal dilatation, dilated congested blood vessels, degeneration of acinar cells, and a few shrunken pyknotic nuclei (Fig. 1C, c). MTX + Gum2 resulted in moderate dilatation of the ducts, periductal fibrosis, mildly dilated congested blood vessels, and minimal degeneration of acinar cells (Fig. 1D, d). The MTX + Gum3 group exhibited minimal dilatation of the ducts, minimal periductal fibrosis, and mildly congested blood vessels (Fig. 1E, e).

Left row, original magnification 100×; right row, 400×; scale bars, 100 μm and 20 μm.

### Caspase 3 immunohistochemical results

**Fig. 2:**
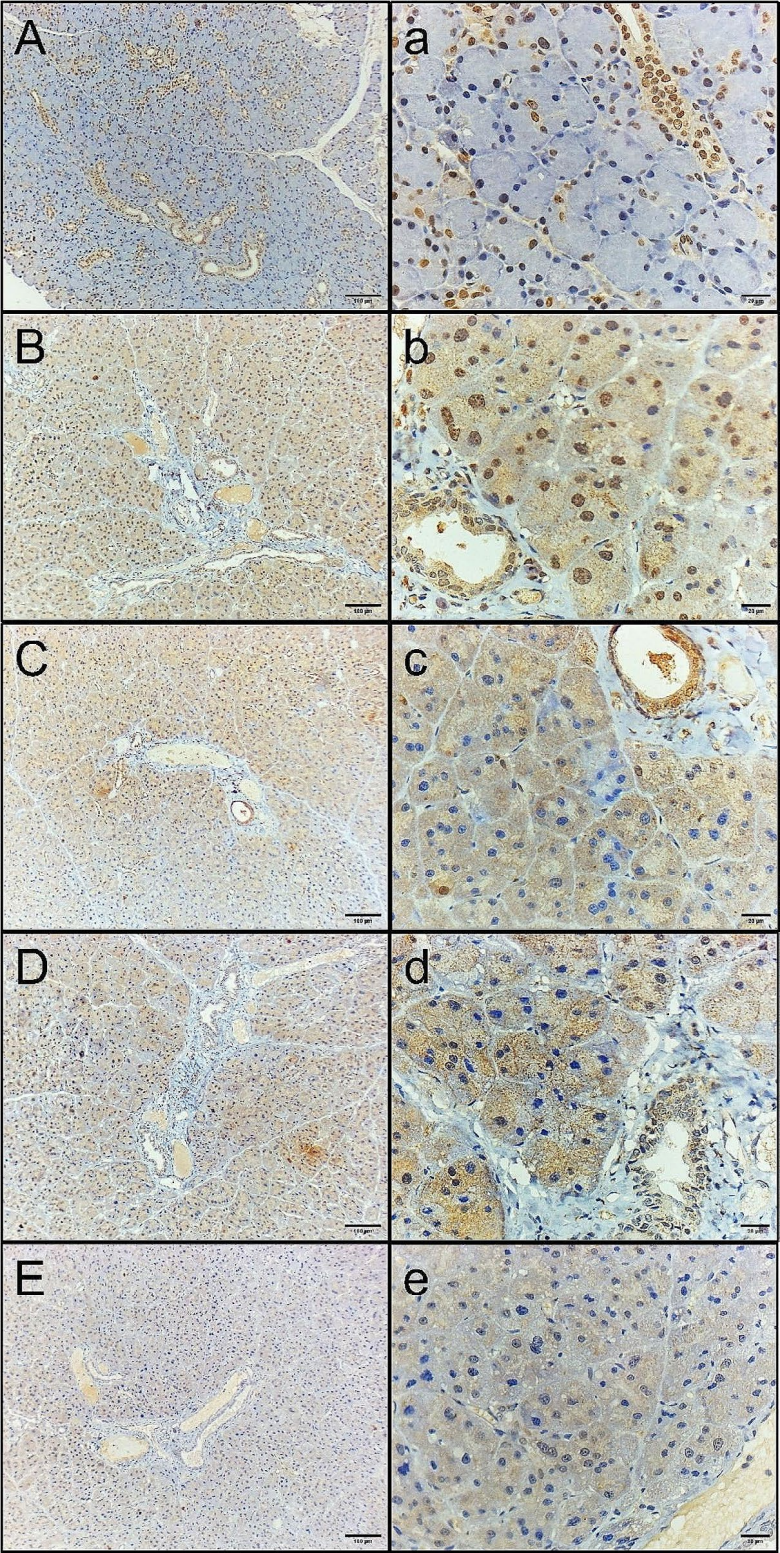
Soft paraffin wax of caspase 3 immunohistochemical staining micrographs of parotid salivary gland sections. **(A, a)** Normal control showed minimal cytoplasmic and nuclear immunoreactivities, especially in the ductal epithelium. **(B, b)** MTX-treated group showing severe cytoplasmic and nuclear **immunoreactivity. (C, c)** MTX + CVM-treated group, showing moderate cytoplasmic immunoreactivity and little nuclear immunoreactivity. **(D, d)** MTX + Gum2-treated group, showing moderate cytoplasmic immunoreactivity and little nuclear immunoreactivity. **(E, e)** MTX + Gum3-treated group, showing mild cytoplasmic immunoreactivity

For this marker, all groups were significantly different from each other according to one-way ANOVA (F ratio = 77.35 and *P* value = < 0.0001). The highest mean value was for the MTX group (54.21 ± 6.90), while the lowest mean value was for the control group (15.75 ± 3.67). The other groups had mean values of 31.09 ± 5.90, 30.76 ± 5.82 and 20.65 ± 3.47 for the MTX + CVM, MTX + Gum2, and MTX + Gum3 groups, respectively. Tukey’s multiple comparisons post hoc test revealed significant differences between the control group and the MTX, MTX + CVM, and MTX + Gum2 groups (*p* < 0.05). On the other hand, there were no significant differences between the control group and the MTX + Gum3 group, and also between MTX + CVM and the MTX + Gum2 group (*p* < 0.05) (Fig. 2) (Table 1).

Left row, original magnification 100×; right row, 400×; scale bars, 100 μm and 20 μm.

### Immunohistochemical results for Ki67

**Fig. 3:**
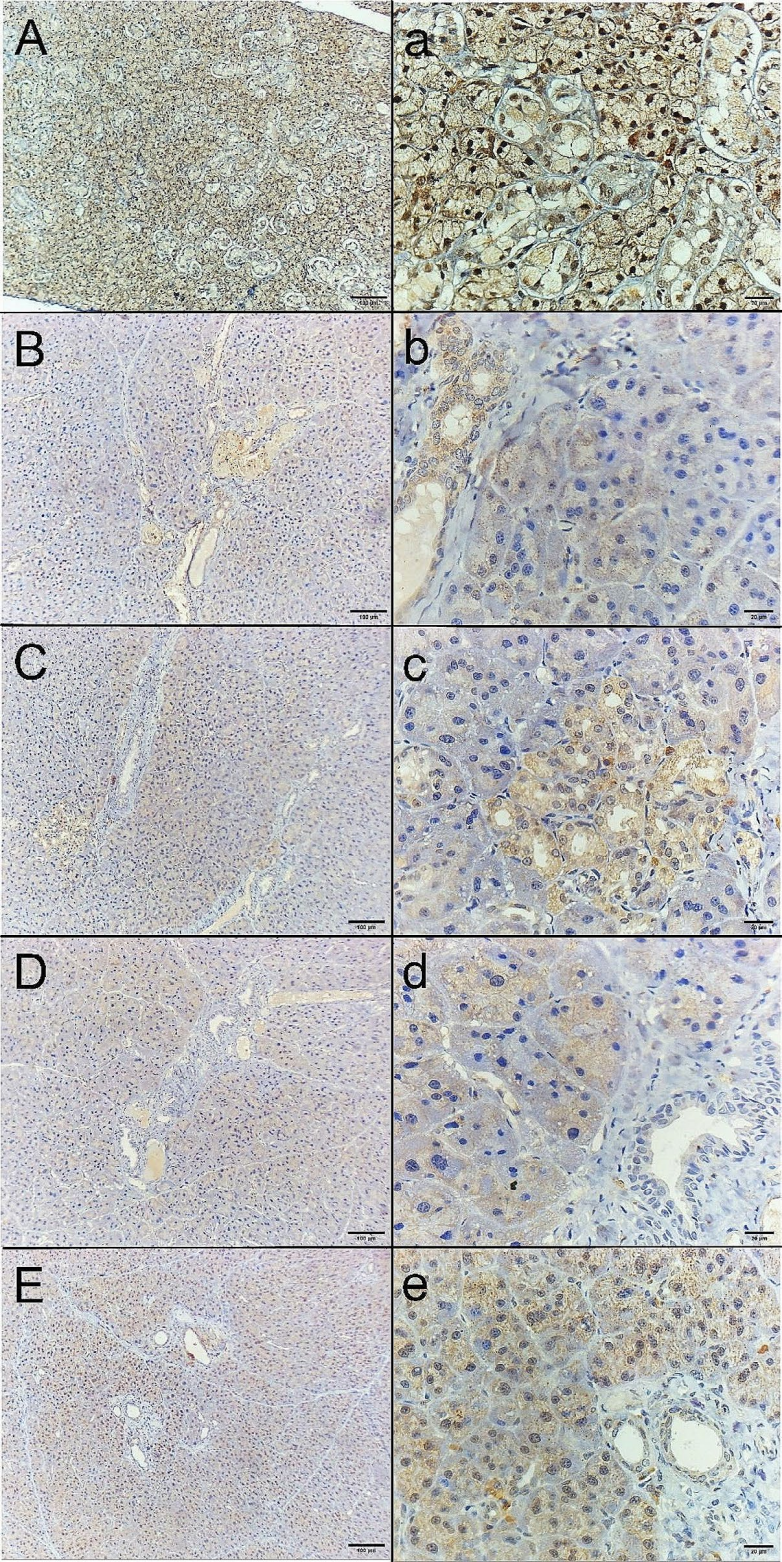
Soft paraffin wax of Ki67 immunohistochemical staining micrographs of parotid salivary gland sections. **(A, a)** Normal control showed strong cytoplasmic and nuclear immunoreactivities. **(B, b)** The MTX-treated group showed weak cytoplasmic immunoreactivity, especially in the ductal epithelium. **(C, c)** MTX + CVM-treated group, showing moderate cytoplasmic immunoreactivity and little nuclear immunoreactivity. **(D, d)** MTX + Gum2-treated group, showing moderate cytoplasmic immunoreactivity and little nuclear immunoreactivity. **(E, e)** The MTX + Gum3-treated group showed strong cytoplasmic and nuclear immunoreactivity

One-way ANOVA revealed significant differences between all groups (F ratio = 129.1 and *P* value ˂ 0.001). The highest value was for the control group (61.70 ± 6.58), while the lowest value was for the MTX group (18.14a ± 5.16). The mean values of the other groups were 34.4 ± 9.27, 48.03 ± 8.40, and 50.63 ± 8.27 for the MTX + CVM, MTX + Gum2, and MTX + Gum3 groups, respectively. Tukey’s multiple comparisons post hoc test revealed significant differences between each group and another group (P ˂ 0.05) (Fig. 3) (Table 1).

Left row, original magnification 100×; right row, 400×; scale bars, 100 μm and 20 μm.

**Table 1:** Descriptive statistics for the different groups of Caspase 3 and Ki67 staining

Groups	Mean ± SD	
	Caspase 3	Ki67
I Control group	15.75 ± 3.67	61.70 ± 6.58
II MTX group	54.21 ± 6.90^a^	18.14 ± 5.16^a^
III MTX + CVM	31.09 ± 5.90^ab^	34.4 ± 9.27^ab^
IV MTX + Gum2	30.76 ± 5.82^ab^	48.03 ± 8.40^abc^
V MTX + Gum3	20.65 ± 3.47^bcd^	50.63 ± 8.27^abcd^
ANOVA (F ratio, *P* value)	(77.35, < 0.0001)	(129.1, < 0.0001)

Values expressed as mean ± SD

Used test: One-way ANOVA followed by post hoc Tukey’s multiple comparison test

a: Significance Vs. control, b: Significance Vs. MTX, c: Significance Vs. MTX + CVM, d: Significance Vs. MTX + Gum2, at *p* < 0.05

## Discussion

GA is considered an antioxidant and anti-inflammatory polysaccharide-based diet. For decades, several communities have recognized it as a hygienic fiber and have used it extensively to alleviate experimental hepatic, cardiac, and renal oxidative and inflammatory injuries in rats [18]. Therefore, the main objective of this study was to evaluate the effect of GA and determine the optimum dose for its desirable effect on the parotid salivary gland.

The histological results of H&E staining revealed intense acinar degeneration in the MTX group. Severe dilation of ducts, periductal fibrosis, and dilation of congested blood vessels were also observed. On the other hand, the MTX + Gum3 group showed minimal dilation in ducts and minimal periductal fibrosis. The other two groups, MTX + CVM and MTX + Gum2, showed mild to moderate dilation in ducts, congested blood vessels, and peritubular fibrosis. In addition, immunohistochemical staining for caspase 3, a marker of apoptosis, was most reactive in the MTX group because it is considered a helpful marker for detecting apoptosis [19]. However, compared with the control group, the MTX + Gum3 group exhibited mild cytoplasmic immunoreactivity, while the other two groups exhibited moderate cytoplasmic reactivity. Finally, Ki67 immunohistochemical staining revealed strong cytoplasmic and nuclear immunoreactivity in the MTX + Gum3 group, as it is considered a proliferative marker [20]. In the MTX group, weak cytoplasmic immunoreactivity was detected, especially in the ductal epithelium, and moderate cytoplasmic and few nuclear immunoreactivities were detected in the other groups.

We believe that this paper is the first to examine the histological effect of GA on the salivary gland. However, Bielfeldt et al. conducted a clinical trial for two groups that had xerostomia—one controlled group and one in which the other had a pastille filled with GA. The authors found a significant increase in the pastille-use group and found GA in the saliva of patients up to 10 min after pastille sucking [21].

Currently, GA is used in traditional medical practice for chronic renal failure (CRF) patients [22]. Therefore, many studies agree with our results, as they used GA to treat CRF induced in rats by adenine injection. The authors found destruction of the glomeruli and renal tubules in groups that consumed adenine only and improvement of renal cortex architecture in groups that consumed adenine with GA [23–25].

Hamid et al. [18] studied the effect of GA on chronic liver injury induced by carbon tetrachloride in rats. The authors used H&E staining and immunohistochemical staining to detect the effect of GA on the injured liver; however, they observed partial infiltration of inflammatory cells and modest centrilobular necrosis in the GA-treated group stained with H&E. The authors used PCNA as an immunohistochemical marker for this study as a proliferative marker [26]. They found that the GA group had greater immune reactivity than did the toxic liver group.

Cevimeline is considered a parasympathomimetic secretagogue used for treating dry-mouth-affected patients, especially those receiving radiotherapy to the head and neck or those with Sjögren’s syndrome (SS) [27]. Therefore, Nakamura et al. [28] used cevimeline as a treatment drug for rats that had SS, and its effect was shown by H&E and immunohistochemistry. The authors found that cevimeline had desirable effects on all major salivary glands. In other studies, normal labial salivary glands were removed from male humans via surgical removal of the tumor in the orofacial region. The samples were immediately cut and placed in a dish with or without cevimine medium for comparison. According to this study, cevimeline causes secretion in the labial glands, and its impact is more physiological than that of pan-muscarinic agonists [29].

Finally, hygienic fiber should be used as a replacement for drugs if it can be effective without any side effects. However, more studies should be performed to obtain more data on either the positive or negative effects of hygienic fibers such as gum arabic.

## Conclusion

Based on the results of this study, we concluded that GA had a desirable effect on the salivary gland and had a greater effect on the antiapoptotic effect than on the proliferative effect. Additionally, the optimal dose of GA was 3 ml/kg.

## Data Availability

The datasets used and analyzed during the current study are available from the corresponding author upon reasonable request.

## Abbreviations

CVM: Cevimeline
GA: Gum arabic
MTX: Methotrexate
H&E: Hematoxylin and eosin
CRF: Chronic renal failure
SS: Sjögren’s syndrome
